# Parental experiences from home visits in the New Families programme: an observational and interview study

**DOI:** 10.1186/s12912-025-03734-1

**Published:** 2025-08-18

**Authors:** Bettina Holmberg Fagerlund, Kari Glavin

**Affiliations:** Faculty of Health Sciences, VID Specialized University, Vinderen, P.O. Box 184, Oslo, N-0319 Norway

**Keywords:** Child health, Home visit, Interpretive description, Parent, Public health nurse, Qualitative study

## Abstract

**Background:**

This study aimed to explore the experiences of parents-to-be and first-time parents with home visits in the New Families programme and how these visits unfolded. The programme was introduced in the City of Oslo in 2019. It offers early support to expectant first-time parents through home visits conducted by public health nurses, starting from week 28 of pregnancy and continuing until the child is 2 years old. The family determines the frequency and content of the visits.

**Methods:**

An interpretive description approach was utilized, adhering to the Consolidated Criteria for Reporting Qualitative Research (COREQ) checklist. Data were collected through eight participant observations and six qualitative interviews. Seventeen parents participated in the study.

**Results:**

Findings indicate that expectant parents had limited knowledge of child health centre services and the public health nurse’s role. Many reported not receiving timely or adequate information about the services or the purpose of the New Families home visits. Despite this, they described the visits as positive experiences, felt treated as equals, and appreciated meeting the nurse who would support them after the birth.

**Conclusions:**

The content and focus of the New Families programme should be clearly communicated to expectant parents before the home visit. Some public health nurses may also benefit from greater clarity about the programme’s purpose and content, which could help avoid misunderstandings among parents. Raising awareness about the purpose of home visits and providing accessible information may encourage more parents to accept the offer of a home visit.

## Background

Becoming a parent marks a significant milestone, and the six weeks after birth can bring significant challenges – often linked to low parental self-confidence. The transition to motherhood begins in early pregnancy and continues until the mother feels she has regained a degree of control over her surroundings. This adjustment period may extend well beyond childbirth [1]. For men, the transition to fatherhood can prompt more profound changes than any other developmental stage [2]. Parents need support and education to help them access reliable information and help related to breastfeeding, effective partner communication, postnatal physical recovery and mental well-being [3]. When a couple transitions to parenthood, their relationship dynamics shift, often resulting in significant strain as they adapt to their new role [4]. According to Feinberg [5] *coparenting* refers to ‘the ways that parents work together in their roles as parents’. It involves the coordination, support – or lack thereof – between individuals who share responsibility for raising specific children. Importantly, the coparenting relationship is distinct from other aspects of the adult relationship; it does not encompass romantic, sexual, emotional, financial or legal dynamics unrelated to childrearing [5]. Improved coparenting is linked to positive child outcomes [6] and research suggests that coparenting support has a greater impact on parental adjustment and postnatal depression than other forms of couple support [7]. Feinberg (2003) also emphasises that coparenting does not require equal parenting roles: The degree of equality is negotiated between parents and shaped by broader social and cultural contexts [5]. Because parents’ approaches are often influenced by their own families of origin, couples may struggle to agree on issues such as how children should behave, how to discipline them, and how to meet their emotional and safety need [6]. Nonetheless, some are able to ‘agree to disagree’ – managing conflict through active and respectful negotiation [5].

A literature review by Wiklund [4] highlights that continuity of care, parental participation, consistent and personalised information, and preparation for parenthood all play a significant role in influencing parents’ sense of security after childbirth. It is also crucial that partners supporting the woman during childbirth feel included and adequately prepared throughout pregnancy, birth and postnatal care. This includes their active participation in interactions within child healthcare settings [8].

In Norway, a core component of child healthcare is the publicly funded municipal child health centres offering a comprehensive, national programme focused on health promotion and illness prevention for families with children from birth to school age [9]. The programme includes 14 scheduled appointments, the first of which is a home visit conducted by a public health nurse, typically 7–10 days after the birth. During this visit, parents receive information about the services offered by the child health centre and can ask questions about any concerns they may have. Topics addressed by the public health nurse in the home visit include parent–child interaction, experiences from childbirth and daily care for the baby (e.g., breastfeeding, vitamins and formula). Additional topics include infant sleep, newborn safety, parental mental health and well-being, social support networks and issues such as smoking, alcohol and drug use, as well as any concerns related to violence, abuse or neglect. The nurse also weighs the baby and measure’s the baby’s head circumference, in addition to examining the baby’s skin, head, eyes, movements and responsiveness [9]. A study by Glavin et al. [10] found that parents usually feel safe during these home visits, as they take place in the home environment and allow for open communication with the public health nurse. The authors emphasise that home visits for families with newborns should serve as a foundation for building a trusting relationship with the family that continues until the child reaches school age [10]. A study by Høgmo et al. [11] found that parents experienced postnatal home visits as positive family experiences. They felt cared for and supported, which contributed to their sense of safety and ability to cope. Subsequent consultations with the public health nurse take place at the child health centre, and most families actively participate in and benefit from this programme [12]. To further support expectant and new parents, many municipalities offer midwifery services in connection with child health centres. As of 2021, midwifery services were available in 99% of all municipalities in Norway [13] and 93% of all 4-year-olds underwent a health examination conducted by a general practitioner at a child health centre [14].

In 2019, a home visiting programme known as New Families (NF) was introduced across all 15 city districts of Oslo as a universal supplement to the national child health centre programme. This initiative was enacted following a decision by the city council [15]. NF developed from a pilot project called the New Mothers Project, launched in 2016 [16]. The New Mothers Project aimed to promote optimal development and growth trajectories for children and provide high-quality counselling to parents in an Oslo city district with the highest child poverty rate in Norway [17]. The pilot phase involved feasibility testing among public health nurses and families to assess the acceptance of, and interest in, a home visiting programme as a complement to the regular child health centre programme [16–18]. The fundamental concept was to provide early-stage support to families in order to reduce the need for more costly secondary interventions in the future, such as child welfare services [17]. The concepts piloted during this phase have been carried forward into the NF programme and are outlined in the implementation manual for the programme [18].

In the NF programme, public health nurses conduct home visits for expectant parents beginning at week 28 of pregnancy and continuing until the child reaches 2 years of age. The programme targets first-time parents-to-be, including couples expecting their first child together or their first child in Norway. It may also be extended to families with multiple children if the relevant child health centre identifies a need for support [16, 18]. NF is rooted in the concept of proportionate universalism [19, 20] meaning that the intervention is both universal and tailored to the individual, with its scope and intensity adjusted to the family’s specific needs. The family determines the frequency and content of the home visits, guided by the public health nurse who acts as a facilitator and supports parental self-efficacy. The programme is grounded in the concepts of *user involvement*, *self-efficacy* and *salutogenesis*. The aim of each visit is to foster mutual understanding between the family and their designated public health nurse regarding the family’s circumstances [16, 18].

## The New Families implementation manual

As stated in the implementation manual [18] in the first home visit, the public health nurse should (a) explain the roles of the public health nurse and the midwife, as well as the purpose of this first home visit, (b) ask about the prospective parents’ own life history and childhood experiences, (c) ask about the prospective parents’ expectations regarding the parental role, (d) ask about the prospective parents’ hopes for their child, (e) initiate discussion about parent–infant communication and interaction and (f) close the conversation by providing information about follow-up after the child’s birth.

The suggested topics for the home visits following the birth of the child are (a) the parents’ participation in society and working/studying, (b) the parents’ relationship, (c) social life and activities, (d) kindergarten, (e) parental fatigue and (f) setting boundaries for the child. In addition, the manual contains guidelines concerning the first telephone call or text message (SMS) to parents, as well as suggestions on how to explain the difference between a public health nurse and a midwife. It also outlines what should be documented from the home visits in the child’s health records [18].

The current study, funded by the Research Council of Norway [21] (registration number: 282167), the City of Oslo and VID Specialized University, aimed to investigate the experiences of parents-to-be and first-time parents related to NF home visits, as well as how these home visits unfolded. The research questions were: [1] What observations can be made about the visit and the dialogue between the public health nurse and the parents-to-be/first-time-parents during the NF home visit [2]? How did the parents-to-be/first-time-parents experience the meeting and their dialogue with the public health nurse at the NF home visit?

## Methods

### Study design

We utilized the interpretive description approach, as described by Thorne [22], since this method is based on a naturalistic orientation and has the potential to generate relevant knowledge for clinical settings. As is characteristic of interpretive description, the inductive analysis of data and the related conceptualisations began alongside data collection, starting from the initial fieldwork [22]. This included both observations and, later, interviews (Table 1). We conducted a qualitative content analysis of the interview data, inspired by Graneheim and Lundman [23].

**Table 1:** Overview of home visit observations (ante- and postnatal), number of interviews, participating parents and their ages

	Observation antenatally (*N* = 6)	Observation postnatally (*N* = 2)	Interview After observation (*N* = 5)	Interview without a preceding observation (*N* = 1)
Mother participating (*N* = 9)	6	2	5	1
Father participating (*N* = 8)	6	2	3	-
Mother’s age (years), median [range]	31.5 [27–40]			
Father’s age (years), median [range]	35 [26–40]			

### Setting and recruitment procedures

Participants were recruited through convenience sampling in three districts in the City of Oslo. We began by consulting the NF programme’s specialist advisor for the City of Oslo, who recommended suitable city districts to contact. We then reached out to child health centre leaders in five districts via phone or e-mail, providing information about the study’s purpose. Three of the five leaders responded positively and shared contact details for five public health nurses willing to take part. The nurses received written information about the study by e-mail and, in turn, informed expectant parents and parents – either orally or by SMS. If parents agreed, the first author was invited to observe a scheduled NF home visit. During the visit, the couple gave consent to be contacted by phone a few weeks later to arrange an interview. One mother agreed to participate in an individual interview without a prior home visit observation.

### Ethics approval and consent to participate

The study was designed and conducted in accordance with the Declaration of Helsinki [24]. When entering the home for the first time, the first author provided oral information about the study and gave the parents-to-be or parents a written information sheet. After reviewing the material, they gave written consent to participate and agreed to be contacted a few weeks later about a follow-up interview. Participation was voluntary, and participants could withdraw at any time without providing a reason. All data were treated confidentially, and participant anonymity was ensured. The study was approved by the Regional Committees for Medical and Health Research Ethics (project number: 27174) and the Norwegian Agency for Shared Services in Education and Research (reference number: 735207). The current study is part of the research project “New Families – Innovation and Development of the Child Health Services in Oslo”, which is registered at ClinicalTrials.gov (Registration Date: 11 November 2019, identifier: NCT04162626).

### Participants

Eight parental couples participated in the home visits. All were cohabiting or married, and all couples were of opposite sexes; their median age is presented in Table 1. All participants were employed outside the home. Mothers went on maternity leave during the final three weeks of pregnancy and after giving birth.

Three of the follow-up interviews included both parents; the remaining three were conducted with the mother only, resulting in a total of nine interview participants (Table 1). Except for two participants from another Nordic country, all were Norwegian. All participants were either expecting their first child or had recently become first-time parents. At the time of the postnatal interviews, the children were between 6 weeks and 3 months old.

### Data collection

Six home visit observations were conducted before the birth, and two took place after the child was born. Five interviews followed these observed visits, and one interview was conducted without an accompanying observation (see Table 1). The home visits typically occurred in the morning, usually beginning at 9 a.m., and lasted between 60 and 80 min. The first author (observer) met the public health nurse outside the entrance to the family’s home. All families lived in neatly refurbished apartments. In five observations, the observer sat apart from the public health nurse and parents, who were seated around a table or in a sofa area. In the remaining three sessions, the observer was invited to sit at the table with the participants and the nurse. The six antenatal visits occurred between one and eight weeks before the due date. The two postnatal visits were conducted two weeks after the birth, with the newborn present (see Table 1).

During the home visits, the first author aimed to minimise disruption to reduce the potential for observer-imposed perspectives. The role was solely as an observer, focused on gathering unstructured observational data through actively listening and taking notes on the implementation and content of the visit. Immediately after each visit, the first author expanded these notes with short memos on the physical setting, participants, activities and interactions, frequency and duration, precipitating factors, organisation and intangible aspects, as presented by Polit and Beck [25]. The first author then summarized this material into a structured observational note for each home visit. Following Polit and Beck [25], we combined observations with interviews to supplement the observational data. The first author conducted the interviews between September 2021 and May 2022. Parents chose the time and location of the interview; two were held in a quiet room at the child health centre, and four took place in participants’ homes. An interview guide was used (Table 2), and interviews lasted an average of 22 min (range: 10–35 min). Recordings were transcribed verbatim by the first author, and sentence structure and grammar were later adjusted to enhance readability.

**Table 2:** Interview guide in english, translated from the Norwegian

Number	Question
1.	Could you share some of your experiences with the home visits?
2.	What expectations did you have regarding the child health centre services prior to the visit?
3.	What information did you receive about the New Families home visiting programme?
4.	How do you perceive the home visits, and how do they meet your needs?
5.	How many times have you had a home visit from the public health nurse?
6.	Is there anything else you would like to add now, at the end of the interview, which we haven’t discussed?

### Data analysis

**Fig. 1:**
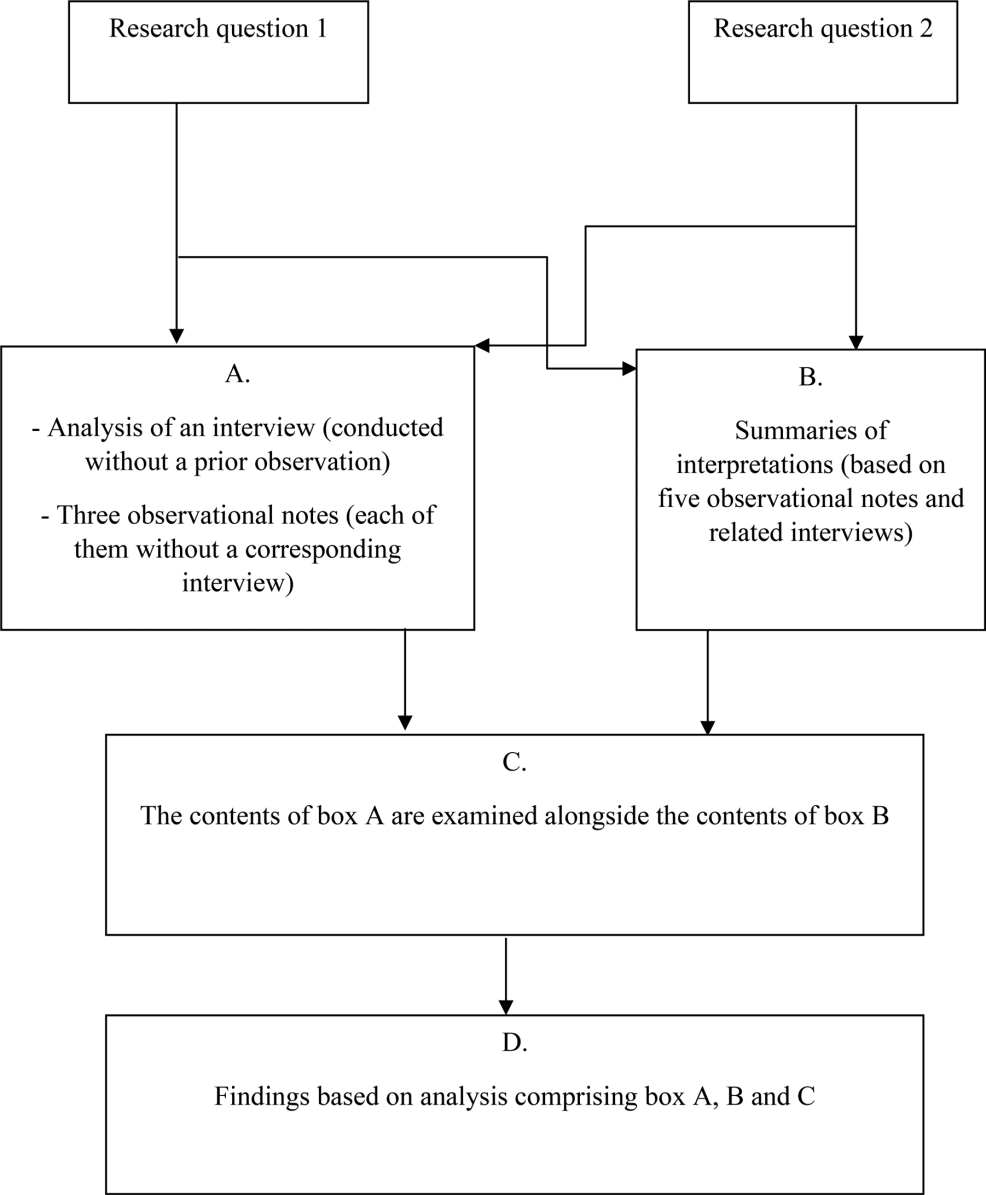
A flowchart of the analysis process

The interviews were analysed using an approach inspired by qualitative content analysis, as described by Graneheim and Lundman [23]. The authors jointly reviewed and discussed the observational notes, enabling the first author to interpret and summarise them in relation to the first research question. When an interview complemented an observation (see Tables 3 and 4), the interview themes were compared with the interpretation of the corresponding observation. Based on this comparison, a new interpretation was formed and summarised by the first author (as exemplified in Table 5). Figure 1 presents the relationships between the components of the analysis based on the research questions and the different data sources. The analysis of the interview, conducted without a prior observation and the observational notes without a corresponding interview were examined alongside the summaries of interpretations based on observational notes and related interviews, guided by the research questions.

**Table 3:** Example of themes from observational note (home visit A)

*Themes based on interpretation*	*Text based on the observational note*
Parents are informed about the purpose of the visit. Meeting took place in a positive, friendly atmosphere.	The public health nurse discusses the purpose of the visit, which is to get to know each other a bit better. The public health nurse also emphasises that new families can receive better guidance later thanks to home visits. The atmosphere is positive, and the parents-to-be express their expectations of soon becoming parents.
Public health nurse appeared hesitant to give practical advice.	After a while, the mother brings up practical matters about what kind of equipment they should have for the child, with some input from the father. It seems that the public health nurse is hesitant to give them clear advice. When the mother enquires about breastfeeding, the public health nurse encourages her to ask as many questions as possible at the hospital but does not provide specific breastfeeding advice.

**Table 4:** Extract from interview connected to home visit A

Meaning Unit	Condensed meaning unit (close to text)	Condensed meaning unit (interpretation of the underlying meaning)	Sub-theme	Theme
No, so we didn’t go to the child health centre that much. Mostly, I went to my GP. I only went to the midwife twice. But she told me about an app, so we went in and had a look at it to find out [about the child health centre]. And then we went to the health centre a few times.	Little contact with the child health centre before the child’s birth, but she received information about an app from the midwife one of the few times that she was there.	Little contact with the health centre service during the pregnancy. Before the birth of the baby, was informed only that there was an app. [Here, one can see information about the child health centre.]	Little information about the child health centre was provided during the pregnancy by the midwife/general practitioner/public health nurse.	No information or preparation ahead of the meeting
We also talked a bit about practical information, then … that you [the mother] sort of had some practical things that we wondered about …	The mother says that she had expected to get answers to practical questions [where to place the changing table/packing a bag for the maternity ward]	The parents were left with unanswered questions. They did not get the impression that the public health nurse wanted to give them answers to many issues they had expected to be able to get answers for.	Parents expect answers to practical questions, which are not the focus of the public health nurse during the home visit.	Avoiding focusing on the couple’s practical concerns

Both authors have extensive experience in public health nursing – as practitioners, researchers, and educators – which provided a relevant pre-understanding. This served as a disciplinary lens that informed interpretation, in line with Thorne’s approach [22]. At the same time, we remained mindful of the importance of avoiding the reproduction of assumptions or taken-for-granted truths, as cautioned by Alvesson and Sandberg [26].

**Table 5:** Summary of interpretations based on contents of Tables 3 and 4

Home visit	Summary
A.	• Parents report having received almost no information before the home visit. • This resulted in parents’ disappointment. • Parents would have liked practical information about breastfeeding and guidance, in advance, for the first two weeks at home with a newborn. • Parents express a need for more engagement with their concerns, rather than a one-sided focus on questions about their own childhood experiences during the pre-birth home visit.

### Findings

This section presents the findings from the analysis, organised into four themes: doubts about accepting the home visit; desire for time to prepare for the home visit; common features of NF programme implementation; and positive atmosphere during the home visit.

### Doubts about accepting the home visit

The home visits were arranged following a phone call or an SMS from the public health nurse to the parents-to-be. The mother was informed by the midwife or general practitioner providing antenatal care that a public health nurse would contact her at some point to schedule a home visit. Some couples explained during the meetings that they had found it challenging to access relevant information about the child health centre and related services prior to the birth.

… I remember when I was first introduced to it by the midwife actually, before the birth – she mentioned that it was something that the child health centre offered. Without kind of saying anything more about what they were offering …. And then I was contacted by my public health nurse, asking if I wanted to …, or if we would like to have a visit then, before the birth. (Mother A)

I didn’t know anything about it. So, we [the parents-to-be] talked about that a bit [beforehand]. We didn’t really know what to expect from the visit. Or what a public health nurse really was. (Mother B)

Several parents described feeling hesitant towards accepting a home visit before the child’s birth when the offer was made by the public health nurse. In three of the interviews, parents explained that they had actively sought advice from acquaintances about whether to accept the offer of a visit and what to expect. Contacts who had experienced a home visit assured them that it had been a positive experience. However, other parents were left confused when those they asked for advice had never heard of home visits being offered before the birth. This led some expectant parents to wonder whether there was a particular reason they had been selected. Despite their doubts, however, they had decided to accept the visit; some parents-to-be then felt stressed, due to their uncertainty and a lack of clarity about how to prepare. They wondered whether they would be assessed on how well their home was prepared for the baby and whether they had acquired the right equipment. Indeed, some parents described feeling as if they were preparing for an examination while awaiting the visit.

We felt a bit like that at first. Because we had no idea what, when this was so early [in the pregnancy] – we felt sort of, ‘We have to tidy up…’, ‘We have to clean …’, ‘Have we bought enough baby things?’ We were thinking ahead a bit, since we were so early in the pregnancy. … So, I feel we were a bit nervous, yes – when we were visited. (Mother D)

Afterwards, they felt relieved when the visit turned out to be pleasant and not at all focused on inspecting them or their home.

After the first home visit, we became more relaxed, actually. I don’t know what did it, but I think it was just the things that were said. That some days are tough, and things like that, and here is information if you’re having a tough time. And that was a helpful thought process. At least I became very relaxed after that home visit. But I think we were a little nervous because we had no idea what this visit was going to be about. (Mother D)

### Desire for time to prepare for the home visit

Because the questions asked during the antenatal home visit were seen as wide-ranging and emotionally demanding about *life’s big questions*, some parents said that they would have appreciated receiving more information in advance – particularly about the purpose and content of the meeting. They found it difficult to be asked such personal questions without preparation. For instance, immediately upon entering the living room and sitting down, one public health nurse asked the expectant father to begin the meeting by answering, ‘Who am I?’ Another asked whether a father had thought about what kind of father he wanted to be. One couple described how their public health nurse initiated a dialogue by asking them to describe their ‘daily contact with the foetus’. During later interviews, the parents reflected that they had found these questions unexpected and quite personal.

It was the first in-depth talk about becoming parents, so I remember I felt a bit like … oh shit, … not so special maybe, but sort of as a clear transition, really. It was the first conversation we had with any professionals about ‘now you are going to become parents, what kind of expectations do you have?’ and so on, yes … In a way, it felt a bit like, for me at least, it was kind of sudden. It was a bit like that, … okay, then I have to think through these things a bit … (Father C)

### Common features of NF programme implementation

A consistent feature of the antenatal home visits was that the public health nurses usually addressed the expectant father first, beginning with questions directed to him before turning to the mother-to-be. All of the public health nurses asked about the parents’ backgrounds, including both positive and negative experiences from their own childhoods. As a result, discussions often involved the parents’ family life growing up, number of siblings, and their relationships with their own parents. The expectant parents were encouraged to reflect on their personal values and the values they hoped to pass on to their child. Afterwards, most parents said that it had been a positive experience to be encouraged to talk about these topics with their partner.

I think it was very good. Because we sort of talked about what expectations we have about becoming parents, and what we … There are things I remember very well when, when she asked, like, what we could take from our own childhood of positive and bad things. My husband and I had a great discussion together after that, too. Though we talked a bit about it that day, we also talked more about it later. Like, what do we expect? Yes … It gave us a bit to talk about, what you expect to bring from your own family, and what you don’t want to bring …. (Mother I)

The public health nurses did not focus on the parents’ practical preparations, such as buying equipment for the baby. If parents raised practical concerns, the nurse would either avoid answering or suggest that they ask someone else. The parents-to-be found this to be somewhat awkward. In the interviews, most explained that they had expected the home visit to include guidance on how to prepare their home and what to expect during and after the birth. They expressed surprise at the lack of response from the nurse on these topics. Indeed, some said that they were slightly disappointed that the visit did not provide practical advice or answers to their concerns about preparing for parenthood.

We also talked a bit about practical information, then … that we sort of had some practical things that we wondered about … but then it was more those things involving principles [about expectations] than practical things [that were in focus]. (Father C)

### Positive atmosphere during the home visit

The public health nurse welcomed the couple and made no distinction in how she included the expectant mother and the father-to-be, inviting both to participate equally in the home visit. Across all visits, the atmosphere was characterised by openness and ease. The parents-to-be consistently demonstrated mutual understanding and shared knowledge of each other’s values and backgrounds. They appeared to be in full agreement, and no doubts or barriers emerged, even when discussing personal aspects of their past. Although the fathers were typically invited to speak first during the antenatal visits, they ensured their partners had ample time to contribute. The couples interacted considerately, taking turns in conversation and showing mutual respect. Even among quieter couples, there was no indication of discomfort or awkwardness.

Parents expressed how pleased they were to get to know the public health nurse who would later follow up with the family through the child health centre. Most had also received helpful support after the child’s birth and particularly appreciated the ease of staying in touch with the public health nurse via SMS.

When we think back to the day we actually had the visit, it was three weeks before our due date, I believe. And it was really nice to see a face, and it was very easy to talk to the public health nurse. It felt very informal and very friendly, I feel. We felt very safe […] The SMS service … I think it’s very good. Even though everyone uses it here, I feel that the SMSs that have been sent, there’s a personal relationship that develops so that you feel safe …. (Mother D)

## Discussion

The study findings are based on the following research questions: [1] What observations can be made about the visit and the dialogue between the public health nurse and the parents-to-be/first-time-parents during the NF home visit? [2] How did the parents-to-be/first-time-parents experience the meeting and their dialogue with the public health nurse at the NF home visit?

A main finding was that participants expressed unfamiliarity with both the services offered by the child health centre and the NF programme. Nevertheless, they still chose to accept the antenatal home visit – often based on encouragement from acquaintances who had had positive experiences. The following discussion is structured around three themes: (a) the importance of being prepared, (b) the content of the visits and (c) building relationships.

### The importance of being prepared

A recent study of the NF home visits found that fathers perceived a hidden agenda behind the visits and experienced a tension between care and control [27]. Similarly, parents in the present study questioned whether there was a specific reason they had been offered a home visit before the birth. Their uncertainty centred on the possibility that the visit might serve as an assessment of their preparedness, which caused stress about how to present themselves and their home. Some sought advice from friends and relatives to help decide whether to accept the visit.

All participants in this study were well established in Norway and had strong Norwegian language skills. It is reasonable to assume that parents with less familiarity with the system or without supportive networks might have remained unsure whether the visit was meant for them. To improve health outcomes and reduce inequities, it may be helpful to strengthen individual health literacy [28] ensuring that all parents receive accessible information about the services offered through child health centres. This includes clarifying that postnatal health checks are a continuation of the care provided during pregnancy.

Solberg et al. [29] found that fathers were uncertain about how often they should attend child health centre visits and about their role during home visits. Many saw themselves primarily as there to support the mother and were unclear about whether they were expected to engage directly with the public health nurse. This lack of clarity sometimes led to reduced participation [29]. Previous studies have also highlighted a general lack of information among expectant parents regarding child health centre services and home visits [30, 31]. In the present study, observations showed that public health nurses always addressed the expectant father first, often beginning with direct and personal questions without prior small talk. One father expressed that he would have appreciated having advance notice about the topics, so he could have prepared. These findings suggest the need for clearer communication from public health nurses about the purpose and content of the visits, starting with their initial contact with prospective parents. Moreover, both parents should be equally invited to participate in consultations and home visits. Existing research also shows that fathers, in particular, desire greater recognition at the child health centre for their role as carers of the newborn [29].

According to Solberg at al. [27], including fathers’ perspectives in the design and delivery of these services may result in a more tailored and inclusive service for the whole family. Another study by Wells et al. [32] found that when fathers received more support, both antenatally and postnatally, from midwives and public health nurses, this was associated with stronger coparenting relationships. Hence, fathers should be encouraged to attend and actively participate in antenatal and postnatal home visits.

National professional guidelines [12] emphasise user involvement in the child health centre. Such involvement helps to ensure the services are tailored to meet users’ needs and improve overall quality. These guidelines also underscore the importance of engaging both parents during home visits [12]. In all observed visits in this study, the public health nurse initiated conversation with the father-to-be first – a finding aligned with prior research showing that nurses view this emphasis on expectant fathers as important when implementing antenatal NF visits [33].

### The content of the visits

The guidelines for the initial antenatal NF home visit recommend that public health nurses explore the prospective parents’ life history, childhood experiences, expectations for their parental role and hopes for their child [18]. These themes – particularly those related to moral values – also appear in the *childrearing agreement* [6], which represents the first step in building a coparenting relationship. According to Feinberg [6], this agreement helps expectant parents develop a mutual foundation for effective cooperation.

An interview-based study by Sæther et al. [31] of first-time parents found that parents wanted public health nurses to proactively offer information about typical challenges during a child’s first year. In contrast, parents in the current study were surprised – and at times unsettled – when the public health nurse did not address practical preparations or respond to questions about childbirth. As they had not received information about the purpose and content of the antenatal visit beforehand, their expectation that they would receive practical guidance in their own home setting is understandable.

A previous study [33] examining public health nurses’ reflections during the implementation of the NF programme found that shifting the focus – from practical concerns to parental expectations and childhood experiences – marked a significant change in their counselling approach. For some nurses, this new emphasis felt unfamiliar and challenging to communicate to expectant parents [33]. Despite this shift, the antenatal NF home visit and the traditional postnatal home visit share important foundational principles. Both are grounded in a health-promoting perspective based on *salutogenesis*, which focuses on factors that support health and well-being [34]. They are also guided by the principle of *proportionate universalism*, offering services to all families while adjusting the intensity and scale of support based on individual needs [19]. In both approaches, a key goal is to strengthen parental self-efficacy: parents’ belief in their ability to succeed in the parenting role [35].

However, the traditional postnatal home visit remains part of the standard child health centre programme, with continued follow-up taking place at the centre itself [12]. In contrast, the antenatal NF visit introduces a new context and focus that requires adjustment – for both professionals and parents.

In the present study, expectant fathers typically spoke at length at the beginning of the meeting, while the mothers spoke only when their partners gave them space to do so. Nonetheless, consistent with previous research [36], all observed visits revealed that the couples had a strong desire for mutual and supportive interaction. Each father adjusted his speaking time to ensure that the mother also had a chance to speak. Couples expressed having shared values and mutual respect, which was particularly evident when difficult topics – such as their own childhood experiences – arose. This demonstrated a level of courtesy that may later support coparenting, as described by Feinberg [5], including fathers’ perspectives can help to create a service that supports both parents and fosters a more family-centred approach [27]. To reduce gender disparities, further research is needed on fathers’ experiences of the content and practice of home visits.

### Building relationships

Parents shared that while some of the topics raised during the antenatal home visit had come up in earlier conversations, they found it meaningful to reflect on them more intentionally with their partner. Revisiting personal themes – such as their own backgrounds, values, and expectations – was described as valuable. A recent study [36] found that first-time parents often seek alignment in their parenting approach, aiming to follow a shared set of rules in raising their child.

In the present study, conversations about parenting expectations rooted in their own childhoods were not typically part of the couples’ daily dialogue. The NF home visit offered a new opportunity to explore these topics, prompting fresh insights and strengthening their connection. Parents described this as a positive and enriching experience.

Research [36] suggests that becoming a parent is a meaningful period of adjustment for couples, making a strong start to both parenthood and coparenting especially important. As the coparenting relationship evolves, challenges are inevitable and require flexibility in daily routines [36]. Supporting the development of strong coparenting early on may also benefit children directly. Altenburger et al. [37] observed that establishing high-quality coparenting behaviours during the antenatal period can increase the likelihood that children are raised in warm, cooperative family environments – an essential foundation for their healthy development.

A narrative review by Cowley and Whittaker [38] highlighted a lack of evaluative research on health visiting practice. In an interview-based study, Solberg et al. [27] found that fathers saw the home visit as a valuable addition to standard child health centre services. They felt the home setting supported the development of a trusting relationship with the public health nurse, though some reported feeling only marginally included in the visit.

These findings raised questions about how different approaches to involving fathers may influence their experiences. In contrast, none of the fathers in the present study reported feeling excluded. On the contrary, they were often addressed first and given time and space to share their thoughts and perspectives as fathers-to-be. This stands in contrast to Solberg et al.’s [27] findings, where fathers described receiving limited attention to their roles as independent caregivers and to their emotional experiences and relationships within the family. Nonetheless, Solberg et al. [24] concluded that antenatal home visits by public health nurses can strengthen fathers’ preparedness and engagement in follow-up care at the child health centre.

In a study by Glavin et al. [10] which focused on home visits conducted by public health nurses within the Norwegian child health centre context, parents reported that the visits felt tailored to their needs and created a space for asking a broad range of questions. These early interactions were experienced as reassuring and valuable. Such support during the transition to parenthood is widely recognized as a key health-promoting and preventive intervention [39]. In particular, research highlights that social support – whether from partners, family members, or professionals – can reduce depressive symptoms in the early postnatal period [40, 41]. Consistent with this, Norwegian interview studies confirm that early support from public health nurses boosts first-time parents’ confidence in their new roles [27, 31].

Parents also appreciated being able to meet the public health nurse before the birth, as this helped to establish a relationship with the person who would later provide follow-up care to the family. This foundation for continuity of care was identified as a key strength of the NF programme in previous studies [31, 33].

### Strengths and limitations

This study offers insights into the NF programme based on observations of home visits and interviews regarding parents’ experiences. However, all parents participating in the study were educated and employed, and all had strong Norwegian language skills. As such, they did not represent the diversity of the broader population of parents in Norway; this could have been addressed by a more targeted recruitment strategy aimed at increasing participant heterogeneity. Nevertheless, the main finding of the study was that these participants, all well-integrated into Norwegian society, expressed unfamiliarity with both the services offered by the child health centre and the NF programme. This finding might transfer to families with different linguistic, cultural, and/or socioeconomic backgrounds.

Moreover, all participating families lived in homes that were conducive to a visit by a public health nurse. This raises the question of whether families in other kinds of living conditions may face additional barriers to accepting such visits. This may partly explain the observed homogeneity in social factors such as education, employment and country of origin. The NF programme is grounded in the principle of *proportionate universalism* and was developed specifically to provide support in areas with higher levels of child poverty. It is therefore important that future studies strive for greater participant diversity to better reflect the general population.

Finally, the data collection took place during a period when infection control measures remained in effect due to the COVID-19 pandemic. These measures limited close contact and delayed data collection, resulting in fewer home visits being conducted during the study period.

## Conclusions

This study highlights a key challenge: Expectant parents often do not receive the information they need about child health centre services and the role of public health nurses prior to using those services. In particular, while the NF programme has introduced new content and a new focus, this is not being clearly communicated to parents in advance. It also appears that some public health nurses are not fully aware of the NF programme’s purpose and content, including the home visits.

To help parents make informed decisions about accepting a home visit, the rationale behind the NF programme must be clearly communicated – especially to the public health nurses, so they in turn can prepare expectant couples. This communication should be based on evidence regarding what the programme includes, its theoretical foundation and the anticipated benefits of the new approach to the home visiting programme.

### Relevance to clinical practice

There remains an information gap that must be addressed to ensure that both public health nurses and parents receive adequate information about the programme. The NF content has been well received by participating parents, which is promising for national implementation.

## Data Availability

The data and materials generated and analysed in this study are not publicly available due to ethical and legal restrictions concerning participant confidentiality. These restrictions were specified in the application to the Regional Committees for Medical and Health Research Ethics and the Norwegian Agency for Shared Services in Education and Research.

## Abbreviations

COREQ: Consolidated Criteria for Reporting Qualitative Research
NF: New Families home visiting programme
SMS: Short Message Service
